# Community Management of Endemic Scabies in Remote Aboriginal Communities of Northern Australia: Low Treatment Uptake and High Ongoing Acquisition

**DOI:** 10.1371/journal.pntd.0000444

**Published:** 2009-05-26

**Authors:** Sophie La Vincente, Therese Kearns, Christine Connors, Scott Cameron, Jonathan Carapetis, Ross Andrews

**Affiliations:** 1 Centre for International Child Health, University of Melbourne and Murdoch Childrens Research Institute, Melbourne, Australia; 2 Master of Applied Epidemiology Program, National Centre for Epidemiology and Population Health, The Australian National University, Canberra, Australia; 3 Menzies School of Health Research, Charles Darwin University, Darwin, Australia; 4 Northern Territory Department of Health and Families, Darwin, Australia; James Cook University, Australia

## Abstract

**Background:**

Scabies and skin infections are endemic in many Australian Aboriginal communities. There is limited evidence for effective models of scabies treatment in high prevalence settings. We aimed to assess the level of treatment uptake amongst clinically diagnosed scabies cases and amongst their household contacts. In addition, we aimed to determine the likelihood of scabies acquisition within these households over the 4 weeks following treatment provision.

**Methods and Findings:**

We conducted an observational study of households in two scabies-endemic Aboriginal communities in northern Australia in which a community-based skin health program was operating. Permethrin treatment was provided for all householders upon identification of scabies within a household during home visit. Households were visited the following day to assess treatment uptake and at 2 and 4 weeks to assess scabies acquisition among susceptible individuals. All 40 households in which a child with scabies was identified agreed to participate in the study. Very low levels of treatment uptake were reported among household contacts of these children (193/440, 44%). Household contacts who themselves had scabies were more likely to use the treatment than those contacts who did not have scabies (OR 2.4, 95%CI 1.1, 5.4), whilst males (OR 0.6, 95%CI 0.42, 0.95) and individuals from high-scabies-burden households (OR 0.2, 95%CI 0.08, 0.77) were less likely to use the treatment. Among 185 susceptible individuals, there were 17 confirmed or probable new diagnoses of scabies recorded in the subsequent 4 weeks (9.2%). The odds of remaining scabies-free was almost 6 times greater among individuals belonging to a household where all people reported treatment uptake (OR 5.9, 95%CI 1.3, 27.2, p = 0.02).

**Conclusion:**

There is an urgent need for a more practical and feasible treatment for community management of endemic scabies. The effectiveness and sustainability of the current scabies program was compromised by poor treatment uptake by household contacts of infested children and high ongoing disease transmission.

## Author Summary

Like many impoverished areas around the world, Aboriginal communities in Australia experience an unacceptably high burden of scabies, skin infections, and secondary complications. Young children are most at risk. Our study investigated scabies in a remote setting with very high rates of skin disease, a high level of household overcrowding, and limited infrastructure for sanitation and preventive health measures. We assessed uptake of scabies treatment and scabies acquisition following provision of treatment by a community-based skin program. In a household where scabies was present, we found that treatment with topical permethrin cream of all close contacts can significantly reduce a susceptible individual's risk of infection. Our findings also demonstrate the challenges of achieving a high level of treatment participation, with limited permethrin use observed among household contacts. This suggests an urgent need for a more practical treatment option. International efforts to reduce childhood morbidity and mortality have demonstrated the efficacy of numerous child health interventions but have also highlighted the deficits in their delivery and implementation. Experiences like this, where the effectiveness of a coordinated local program delivering an efficacious intervention is hampered by poor treatment uptake and ongoing transmission, are an important and timely message for researchers, program managers, and policy-makers.

## Introduction

Skin infections are a significant cause of morbidity in disadvantaged settings around the world [1],[2]. Scabies and pyoderma are endemic in many Aboriginal communities in northern Australia. These conditions cause local morbidity and contribute substantially to clinic workload and costs [3]. Moreover, the primary bacterial pathogen underlying most pyoderma in these communities is Group A *Streptococcus* (GAS), which can cause a myriad of debilitating secondary complications [4], including acute nephritis. Recent evidence also suggests a link between GAS skin infection and rheumatic fever, and consequently rheumatic heart disease [5]. Post-streptococcal disease rates in Aboriginal Australians are among the highest in the world [6]. Scabies is thought to underlie the majority of bacterial skin infections in these communities [7], thus controlling scabies is critical to improving skin health and reducing the burden of GAS secondary complications. Scabies is a disease that often accompanies poverty, with high prevalence being consistently associated with crowded living conditions [8]–[10].

Previous experience suggests community-based mass-treatment approaches are likely to be the most effective for control of scabies and skin sores in remote Aboriginal communities [1], [11]–[13]. Similar models are used to control endemic parasitic and other diseases around the world [14]–[16].

Substantial reductions in the prevalence of scabies in endemic settings have previously been described with mass community treatment using oral ivermectin [17] and topical permethrin [18],[19]. However, sustainability has been difficult to achieve [2],[12], particularly where there is high mobility between communities and households. A high level of community participation is critical to the success of community-based programs such as this. To maximise treatment uptake during both mass distribution and routine screening, the treatment must be acceptable and feasible in the setting to which it will be applied [20].

A skin health program incorporating mass annual distribution of permethrin cream, routine scabies screening and treatment at the clinic and in homes, has been operating in the remote Aboriginal communities of the East Arnhem region of the Northern Territory since 2004. Over this time, scabies prevalence amongst children in the region remained unchanged at 13% [21].

Due to the ongoing scabies burden in these communities we aimed to assess levels of treatment uptake among households in which one or more members was seen with scabies during routine screening. We also sought to investigate acquisition of scabies among household members during the month after the initial visit.

## Methods

### Setting and population

The study was conducted in two Aboriginal communities participating in the East Arnhem Regional Healthy Skin Program in remote northern Australia. A review of presentations to the community health clinics in 2006 revealed that 63% of infants had been diagnosed with scabies and 69% with skin sores in the first year of life [3].

### East Arnhem Healthy Skin Program

The skin program included annual mass community treatment for scabies (a designated “Healthy Skin Day”), combined with ongoing screening, treatment and follow-up of children aged <15 years in each community. On Healthy Skin Day, all individuals were encouraged to apply topical permethrin 5% (Lyclear), which was distributed directly to all households in the community. Residents were advised to apply the cream all over the body and leave it on overnight or for a period of at least 8 hours. Healthy Skin Day was held within the month prior to the commencement of data collection for this study.

The East Arnhem program included routine skin assessments of children under 15 years throughout the year, which aimed to identify and treat reinfestation arising after the mass community treatment. These were conducted at the community clinic, during school screening programs and on home visits. Where a child with scabies was identified, permethrin cream was provided for all household members. Locally-relevant resources were produced and used by local workers to teach people about scabies and skin health during the assessments. The program team included doctors, nurses, dermatologists, Aboriginal health workers and locally employed community workers. A Darwin-based team visited regularly to support the local community workers. The program had been operating in these communities for 2.5 years when this nested study commenced.

The nested study population was households where one or more children had been diagnosed with scabies during routine home visits. Data were collected between December 2006 and June 2007. Informed written consent was given by all participating households. This study was approved by the Human Research Ethics Committee of the Northern Territory Department of Health and Menzies School of Health Research (Approval number 04/11).

### Study procedures

A member of the Darwin-based team visited each community every two weeks during the 6-month study period to assist the community workers in recruitment and follow-up of households.

Children with scabies were identified during home visits for routine skin screening undertaken as part of the existing skin health program (Day 0). The first child from a given household seen with scabies became the index child for that household. In line with normal skin program practice, the mother/carer of this child was provided with permethrin cream for all household members, together with standardised verbal instructions for cream use. Local guidelines for scabies treatment indicate that all household members should be treated, regardless of individual scabies status. Household members included the index case and all individuals considered by the index child's mother/carer to be living in the house at that time, as occurs during normal skin health program procedures. All household members other than the index case are referred to as the index cases' household contacts. Instructions were given for the index child and all other household members to use the cream that night. As noted, provision of treatment was according to normal skin health program procedures, and was not dependent on participation in this study.

The date of birth, sex, scabies and skin sore status of each household member was recorded. Household members with skin sores were referred to the clinic for treatment.

Recommendations regarding environmental measures to eradicate scabies from the household were also provided, which included washing all clothing and linen. Participation in these activities was not necessary for an individual or household to be considered to have participated fully in treatment. Evidence indicates that the role of fomites in transmission of scabies is minimal [22],[23]. However we consider these environmental recommendations to be positive public health messages, particularly in a highly overcrowded setting such as this.

On Day 1 we revisited the home and asked the mother/carer which household members had used the treatment. Treatment uptake was defined at the individual and household level. For an individual, we accepted either self-report or report from the primary carer that the household member had used the cream. Complete household treatment uptake occurred if all household members were reported to have met the definition for individual treatment uptake. If household treatment participation was incomplete, the mother/carer was asked if she could say why household members had not used the cream.

Subsequent home visits were undertaken on Days 14 and 28 to screen household members for scabies and skin sores. Additional permethrin cream was provided as required at these visits. Utilisation of cream provided after Day 1 was not assessed.

### Diagnosis of scabies and skin sores

Scabies was diagnosed clinically by specifically-trained staff, using accepted criteria and based on the nature and distribution of characteristic scabies lesions [24],[25]. This is the standard and widely accepted approach to scabies diagnosis in an endemic setting such as this [17],[26],[27]. Skin sores were also diagnosed clinically, based on the presence of any crusted, purulent or dry sores. Where a household member was not present during the home visit, we asked the primary carer in the household to report whether the individual had scabies and/or sores. Findings are described according to whether a trained Healthy Skin Worker (HSW) screened the individual or if skin status relied on family member report.

When measuring scabies acquisition (defined as an individual who was scabies-free at Day 0 and had a clinical diagnosis of scabies within 28 days) we classified incident cases as either confirmed or probable. “Confirmed” scabies acquisition required an individual to be seen by a HSW both at baseline (with a clinical diagnosis as scabies-free) and again at a follow-up (clinical diagnosis of scabies) Report of scabies acquisition by a family member was considered “probable”. These were individuals who were: a) clinically diagnosed as scabies-free at baseline by a HSW and then reported to have scabies by a family member at the follow-up visit or; b) were not seen by a HSW at baseline but were reported to be scabies-free by a family member and were subsequently diagnosed as scabies by a HSW at follow-up or were reported to have scabies by a family member at the follow-up visit.

### Analyses

Data were analysed using Intercooled Stata 9 (Stata Corporation, College Station, Texas, USA). Comparisons of continuous variables across two levels of a binary variable were conducted using the Mann Whitney U test. For comparison of proportions between two binary variables, relative risks and 95% confidence intervals (CI) were calculated. Chi square test or Fisher's Exact test were used to test the significance of associations where appropriate.

To assess the relative contribution of independent (explanatory) variables to individual treatment uptake, logistic regression analysis was implemented using the method of marginal models estimated using Generalised Estimating Equations (GEE) with information sandwich estimates of variance. GEE was used due to the potential for clustering among households. An exchangeable correlational structure was used. These analyses were performed in Stata using the xtgee command.

In exploring differences between susceptibles who did and did not acquire scabies over the one-month follow-up period, GEE was also employed given the potential for clustering by household. Since subgroups contained small numbers of individuals, there was insufficient power to test all explanatory variables by multivariate GEE model. Therefore, simple (univariate) GEE was performed for each independent variable of interest. This provided a measure of the significance of the association between acquiring scabies and each independent variable while controlling for the potential influence of household clustering.

## Results

### Description of sample

Forty households participated, involving 596 individuals (40 index children, 556 household contacts). Median household size was 15.5 persons (IQR 12, 20). At baseline (Day 0), a median of 23.6% (IQR 11.0, 44.7) of individuals in each household had scabies as determined by either HSW screening or family member report (Table 1). All households in which a child with scabies was identified during normal skin program activities agreed to participate in the study.

**Table 1:** Description of participating households and individuals in each community.

	Community A	Community B	p	Total
**Households**	**N = 18**	**N = 22**		**N = 40**
Persons per household∧ *	14.0 (12, 18)	18.0 (14, 21)	<0.001	15.5 (12, 20)
Crowding∧ (persons per bedroom)	4.3 (3.3, 5.3)	4.5 (3.5, 5.3)	0.510	4.3 (3.5, 5.3)
% householders under 5 years∧	14.4 (9.6, 22.0)	20.5 (14.9, 24.7)	0.134	17.9 (12.3, 24.1)
Household scabies burden at Day 0∧ * (% householders with scabies)	33.3 (17.1, 50)	16.2 (9.1, 26.3)	<0.001	23.6 (11.0, 44.7)
% householders screened at Day 0∧	47.7 (27.6, 67.2)	44.5 (33.3, 66.3)	0.946	46.4 (31.0, 67.1)
**Individuals**				
***Index children***	**N = 18**	**N = 22**		**N = 40**
Age (years)∧	3.9 (1.1, 10.8)	2.7 (0.7, 5.5)	0.265	3.4 (1.0, 5.9)
Sex (% male)	50% (n = 9)	50% (n = 11)	1.0	50% (n = 20)
Scabies at Day 0&	100% (n = 18)	100% (n = 22)	1.0	100% (n = 40)
***Contacts***	**N = 221**	**N = 335**		**N = 556**
Age (years)∧	19.0 (7.8, 33.5)	22.0 (6.5, 33.2)	0.973	20.2 (7.4, 33.4)
Sex (% male)#	50.5% (n = 110/218)	51.4% (n = 165/321)	0.830	51.0% (n = 275/539)
Scabies at Day 0# * +	21.7% (n = 30/138)	12.7% (n = 35/276)	0.017	15.7% (n = 65/414)

**∧:** Values are median (IQR).

# Individuals with unknown status (missing data) have not been included – denominator for calculation of percent is individuals with known values.

*: Significant difference between Community A and Community B (p<0.05), Wilcoxon Rank Sum test for continuous variables, Chi Square for categorical variables.

& All cases diagnosed by Healthy Skin Worker (HSW).

**+:** In Community A, 13 cases diagnosed by HSW, 17 by family report. In Community B, 22 cases diagnosed by HSW, 13 by family report.

### Treatment uptake

#### Index children

**Figure 1:**
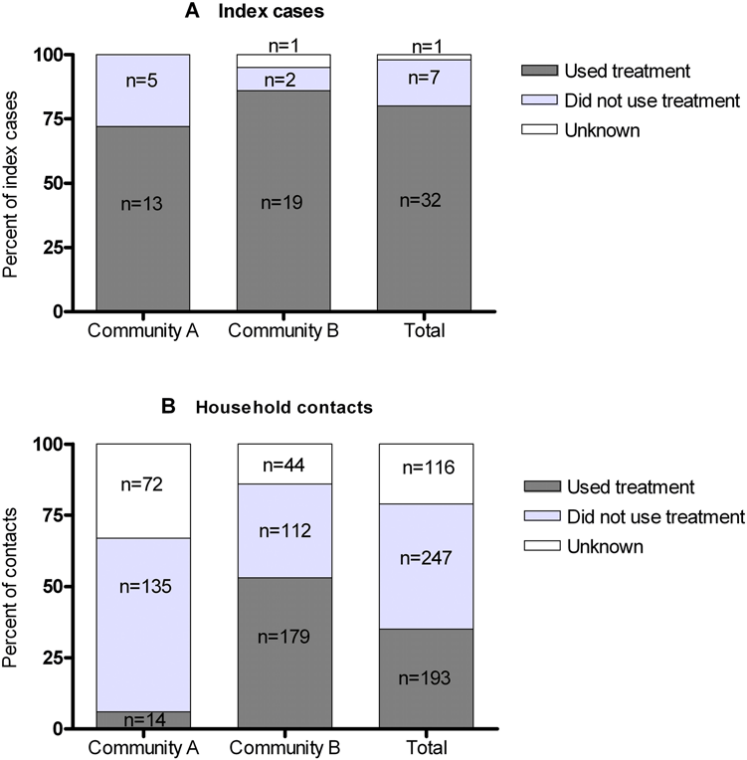
Reported treatment uptake among index children (A) and household contacts (B) in each community and in total. Denominator used in calculation of percentage includes individuals with unknown status.

Of the 40 index children, 32 (80%) were reported to have used the cream. A similar level of treatment uptake among these children was reported in both communities (RR 1.3, 95%CI 0.91, 1.72) (Figure 1).

#### Household contacts

Treatment uptake was established for 440 of the 556 household contacts (79%). Most contacts (247/440, 56%) did not use the cream. Household contacts in Community B were over 6 times more likely to report treatment uptake than those in Community A (RR 6.5, 95%CI 3.9, 10.9) (Figure 1).

#### Complete household treatment uptake

**Figure 2:**
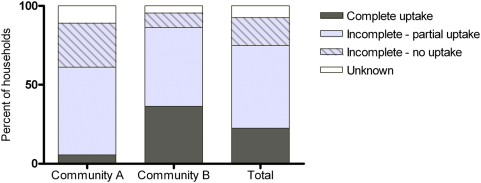
Percentage of households reporting complete or incomplete treatment uptake among households members in each community and combined.

Overall, household treatment uptake information was available for 37 of the 40 households. Most households (28/37, 76%) did not have complete treatment uptake amongst all household members. In almost 20% of households there was no treatment uptake by any individual (Figure 2).

In Community B, complete treatment uptake amongst members was reported by 8 of 21 households (38%). In contrast, there was only one household in Community A (1/16, 6%) where complete treatment uptake was reported for all household members. Although not statistically significant, the point estimate for complete treatment uptake was 6 times higher in Community B than Community A (RR 6.1, 95%CI 0.85, 43.9). Similarly, there was a higher proportion of households with no treatment uptake reported for any household member in Community A, 31% (5/16), compared to Community B, 9.5% (2/21) (Figure 2).

Barriers to treatment uptake were reported by the mother/carer in 24 of the 28 households with either incomplete or no treatment use. These barriers included the treatment not being a priority (n = 15, 54%), that individuals without scabies felt they did not need to use the cream (n = 11, 46%) and that individuals didn't want to use the cream as it was uncomfortable, hot and sticky (n = 10, 42%).

#### Factors associated with treatment uptake

Households reporting complete treatment uptake had a significantly lower median household scabies burden at initial assessment (proportion of household members with scabies) than those reporting incomplete treatment uptake: 11% vs. 26%, Z = 1.97, p = 0.04. In contrast, household crowding (persons per bedroom) was similar among households reporting incomplete or complete treatment uptake (4.0 vs. 3.5 persons per bedroom, respectively) (Z = 0.67, p = 0.50).

The odds of treatment uptake was 2.4 times greater among household contacts with scabies compared to those without (OR 2.4, 95%CI 1.1, 5.4) (Table 2). By contrast, the odds of treatment uptake was significantly less among males (OR 0.6, 95% CI 0.42, 0.95), individuals from households with a high scabies burden (OR 0.2, 95% CI 0.08, 0.77), and individuals from Community A (OR 0.2, 95% CI 0.05, 0.69). There was no evidence that age (OR 1.0, 95% CI 0.99, 1.01) or household crowding (OR 1.2, 95% CI 0.4, 4.0) influenced treatment uptake.

**Table 2:** Multivariate GEE model of factors associated with individual treatment uptake among household contacts.

	OR	95% CI
Scabies*	2.4	1.10, 5.43
Sores	2.3	0.88, 5.86
Age	1.0	0.99, 1.01
Household crowding	1.2	0.36, 4.04
Sex (male)*	0.6	0.42, 0.95
Community*	0.2	0.05, 0.69
Household scabies burden*	0.2	0.08, 0.77

*: Significant association with individual treatment uptake.

OR odds ratio, CI confidence interval.

### Acquisition of scabies

#### Household contacts at risk of scabies

In total, there were 349 individual household contacts who did not have scabies at baseline (Day 0). Of these, 164 were lost to follow up. Among the remaining 185 susceptible individuals, there were 9 confirmed incident cases of scabies and 8 probable incident cases over the 4-week follow-up period. Including both confirmed and probable cases, the acquisition rate was 9.2% (17/185). This was 5 times greater (RR 5.2, 95%CI 2.2, 12.2) in Community A (32%, 7/22) than Community B (6%, 10/163).

#### Remaining scabies-free

Among the individuals who did not have scabies at baseline, remaining scabies-free was not associated with individual treatment use (OR 1.3, 95%CI 0.4, 3.9, p = 0.6); however the odds of remaining scabies-free was almost 6 times greater among individuals belonging to a household where all people reported treatment uptake (OR 5.9, 95%CI 1.3, 27.2, p = 0.02). Those who acquired scabies were significantly younger (median 4.8 years, IQR 2.3,7.4) than those who did not acquire scabies (median 25.8 years, IQR 11.9,38.7) (Z = 4.8, p<0.0001). There was no evidence that household crowding (OR 1.0, 95%CI 0.6, 1.7, p = 0.09) or household scabies burden (OR 1.0, 95%CI 0.9, 1.0, p = 0.7) were associated with likelihood of acquiring scabies among these individuals.

The GEE analyses of confirmed and probable incident cases provides adjustment for potential clustering by household. Whilst small numbers preclude a similar analysis amongst the confirmed cases alone, we have undertaken a secondary analysis (without controlling for household clustering) to compare the risk factors for the “confirmed” scabies cases against those household contacts who did not acquire scabies. Here, consistent with the findings from the GEE analysis, we found those with “confirmed” scabies acquisition were more likely to come from a household that did not have complete treatment uptake and were much younger than those household contacts who did not acquire scabies (Table 3).

**Table 3:** Comparison of confirmed incident cases and non-cases.

	Acquired scabies (N = 9)	Did not acquire scabies (N = 168)	
Individual treatment uptake	Yes 62.5% (5/8)	Yes 63.0% (102/162)	RR 1.2, 95%CI 0.33, 4.64
Belongs to household with *complete* treatment uptake*	Yes 0% (0/9)	Yes 46.1% (76/165)	RR, 95%CI – N/A; p = 0.005
Age∧ (years)*	5.8 (11.3,2.3)	25.8 (38.7,11.9)	Z = 3.2, p = 0.001
Household crowding∧	4.0 (5.3, 3.5)	4.0 (5.3, 3.5)	Z = 0.09, p = 0.930
Household scabies burden	8.3 (26.7, 5.3)	15.8 (25.0, 7.7)	Z = 1.05, p = 0.295

**∧:** Median (IQR).

*: Significant difference between confirmed incident case and non-case.

Note where there are individuals with unknown status, % has been calculated based on total individuals with known status.

N/A -point estimate and confidence intervals cannot be calculated due to nil value.

A comparison of susceptible individuals lost to follow up (n = 164) and those for whom scabies status over the 4-week period was obtained (n = 185) revealed two significant differences between these groups. Individuals were significantly more likely to be followed-up if they belonged to households with complete treatment uptake (43.1% vs. 14.9%, RR 1.75, 95%CI 1.5,2.1), and if they were reported to have used the treatment themselves (62.2% vs. 39.6%, RR 1.5, 95%CI 1.2, 1.9).

## Discussion

Our study is the first to investigate levels and determinants of treatment uptake in a scabies endemic setting. Treatment uptake among index children was over 70%, suggesting that it is possible to achieve high levels of individual treatment use. However, treatment uptake among the household contacts of these children was poor and there were very few households in which all members were reported to have used the treatment. We are likely to have achieved a higher level of uptake in this study sample than would otherwise have occurred, given that householders were aware we would return the following day. Routine Healthy Skin program procedures do not include this home visit the day after treatment provision. In addition, self-reported treatment uptake is likely to be subject to reporting bias, which may also have resulted in an overestimation of treatment uptake. Therefore, even under circumstances of increased motivation to use the treatment and potential over-reporting of uptake, observed rates of treatment use were still very poor amongst household contacts. This low participation in treatment has important implications for the likelihood that sustained reductions in scabies burden can be achieved with the community-based model employed here.

Our findings support current recommendations for universal treatment of close contacts where scabies is present, with the odds of scabies acquisition being greatest among young children and individuals in households with incomplete treatment uptake. Even in an endemic setting characterised by high mobility between households, universal treatment among family members in households where scabies was present significantly reduced the likelihood of acquisition among susceptible individuals. This is critical to protect young children, who are most at risk of infection. However, we have also demonstrated that the current approaches to achieving universal treatment are neither feasible nor effective in this setting. Although there was an established community-based control program in the two communities in which this study was conducted, we observed a very high level of secondary transmission. While the risk of secondary transmission remained unacceptably high in both communities, it was five times higher in Community A than Community B. The former had both a comparatively higher household burden of scabies and lower levels of treatment uptake amongst household contacts, which helps to explain the differential.

Overall, reported treatment uptake was poorest in settings with a high scabies burden. This was observed at both the community level and at the household level. As noted, Community A had a higher median household scabies burden compared to Community B, and significantly less treatment uptake. In addition, irrespective of community of residence, an individual from a household with a high scabies burden was less likely to use the treatment than an individual from a household with a low scabies burden. The depressing reality for many households in these communities may well be that scabies has become part of life. At the individual level, treatment failure, or failure of others to adequately treat, creates an ongoing cycle of scabies transmission within both the household and the community. This may well contribute to the likelihood that those households with high scabies burden become increasingly less likely to treat.

A number of community-based initiatives have documented a sustained reduction in scabies burden in endemic settings using topical permethrin. In Panama, directly observed permethrin treatment of all inhabitants of an island community, together with ongoing surveillance and treatment of new cases, resulted in a significant reduction in scabies prevalence [18]. Similar results have previously been reported in Australian Aboriginal communities without the requirement of directly observed treatment [12],[13]. That the achievements of previous programs have not been observed here may reflect community-specific factors, such as social or environmental characteristics, which may not be generalisable to other communities or regions. Indeed, even in the current study we identified marked differences in treatment participation and household scabies burden between the two participating communities. Furthermore, given the low levels of treatment participation observed here, it seems unlikely that complete participation would have been achieved during the mass community treatment event that took place in the weeks prior to our study. If a substantial and rapid reduction in community scabies prevalence could be achieved through complete participation in mass treatment, the subsequent management of new cases at the household level may be a more feasible and effective approach. However, the high level of movement within and between households and communities would still present significant challenges to maintaining a low prevalence of scabies.

The difficulty in achieving a sustained reduction in scabies burden in these settings has previously been recognised [2],[12],[18]. In Australia and many other countries, topical preparations such as permethrin cream are the only approved treatment for community management of scabies. The practicality of topical treatment for the community management of endemic scabies has been questioned [9],[25]. Indeed, several characteristics of the treatment and setting give reason to doubt that a sustained reduction in scabies prevalence can be achieved with this approach. Environmental factors make total-body topical treatment impractical. These factors include the large number of people in each house, high heat and humidity, limited opportunities for privacy to apply the cream [1], and poor infrastructure for washing it off [28]. When complete treatment uptake does occur, there is a strong likelihood of rapid reinfestation due to the high prevalence of scabies, overcrowding and frequent movement between households and communities. A realistic consequence of this is low motivation to repeat the treatment process.

The likelihood of individual participation in treatment for the community management of endemic parasitic infection has been linked to expected personal benefit and expected personal cost [29]. It seems likely that the low level of uptake observed here, particularly where there is a high burden of scabies, is symptomatic of low motivation to participate in a treatment regime that is onerous (high time and inconvenience) and has been seen to have limited effectiveness (low personal benefit). This is supported by the reasons cited for non-participation in treatment, the most common of which was that using the treatment wasn't a priority. Many also reported the inconvenience and unpleasantness of the treatment to be a key barrier to use.

Of additional concern is the potential for the development of drug resistance [30],[31] when such long-running community disease control programs achieve only limited participation and disease reduction. Concerns regarding mite resistance to permethrin have recently been described in a number of Aboriginal communities in northern Australia [32],[33]. Thus it is possible that even if greater levels of treatment participation could be achieved, resistance to this treatment may undermine any potential impact on disease burden.

These findings demonstrate an urgent need for a more suitable treatment for scabies to reduce the burden in endemic settings. Oral ivermectin has demonstrated success in the community management of endemic scabies [17],[26],[27]. For example, in the Solomon Islands, mass ivermectin administration to all residents of a small number of islands, combined with treatment of any individuals subsequently entering or returning to the communities, reduced scabies prevalence from 25% to <5% after four months, and was subsequently sustained at <1% [17].

Ivermectin is also widely used in community control of other parasitic infestations. Since 1987, it has been used extensively in community-based mass treatment programs in Africa and Latin America to control endemic onchocerciasis [34]. The Global Programme to Eliminate Lymphatic Filariasis recently reported that between 2000 and 2007, 149 million treatments of ivermectin had been given through community-based mass drug administration in 12 African countries [35]. Ivermectin is also effective against other parasitic infestations that can occur in high-scabies burden settings, such as strongyloidiasis [26], which is endemic in many Australian Aboriginal communities [36].

Ivermectin is not currently approved for the mass community management of scabies in Australia. Notwithstanding, a growing body of literature indicates it is safe and effective when used in mass drug treatment programs and is ideally suited for use in the community as it is a single oral dose that is easily administered. In 2003, it was estimated that 6 million people worldwide had taken ivermectin for various parasitic infestations with no serious drug-related adverse events reported [37]. Contributing to the safety profile is the accumulation of non-event data among pregnant women [38]–[40].

While ivermectin presents a viable alternative for the management of scabies, especially where compliance with topical treatment is improbable or impractical [34],[37], the provision of a more practical treatment alone is unlikely to completely resolve the low treatment participation seen here. Many households cited reasons for non-participation that may not be readily resolved simply with a more practical treatment. For example, there is an enduring perception that individuals without scabies do not need to be treated. Factors that have been identified as important for treatment participation and program success in mass ivermectin administration include strong community education and awareness-raising, engaging well-trained health workers who are trusted and respected by the community, and community involvement and ownership in the program [41]–[43]. It will be critical to consider such factors in the implementation of any initiative to reduce the burden of scabies in these communities, and to work together with communities to better understand household and individual barriers to program participation.

Our study has several limitations that are inherent to research in this setting. With large and transient family groups, the concept of a household unit is tenuous and follow-up of individuals is compromised. While complicating data collection, this also further highlights the challenge of infectious disease management at the household level. In our study, almost half of the susceptible individuals were lost to follow up. We must therefore consider the possibility that those individuals who were followed up represent a biased sample of the population at risk. Those who were lost to follow up were significantly less likely to belong to a household in which all members used the treatment, and less likely to use the treatment themselves. This suggests that we have missed a segment of the susceptible population that may indeed be at a higher risk of acquiring scabies, and we may therefore have underestimated acquisition.

Where a household member was unwilling or unavailable to participate in skin screening, a family member reported scabies status for that individual. Ideally, all household members would have been available for skin screening. The presence of itching and visible skin lesions in a household where scabies is known to be present has been reported to be highly specific [11]. Notwithstanding, here we have distinguished between scabies cases diagnosed by a trained worker (confirmed case) and those reported by a family member (probable case) in order to mitigate any potential impact of this on the validity of our data.

There is limited evidence for the effective, long-term management of scabies in high prevalence areas [44]. Achieving a significant reduction in infectious disease burden in an endemic setting requires a high level of treatment coverage among those exposed to the disease. Here we have demonstrated a lower likelihood of scabies acquisition when all close contacts participate in treatment. This emphasises the importance of all close contacts of scabies cases being treated, whether symptomatic or not. This is recommended in all settings where scabies is present. However, achieving this high level of participation in treatment can pose a considerable challenge. Our findings indicate that the long-term management of scabies with topical permethrin has not been effective, as evidenced by low rates of treatment uptake and ongoing transmission. These results support the notion that, in controlling infectious diseases such as scabies at the community level, it is critical that the treatment be appropriate, acceptable and feasible in the setting to which it will be applied. It is also fundamental to recognise that the eradication of scabies and other infectious diseases in these settings cannot be achieved by treatment alone. To realise a significant and sustained reduction in disease burden requires that the underlying environmental and social conditions that promote such poor health be addressed.
